# Harmful effects of propofol? The Editor’s standpoint

**DOI:** 10.1186/s13054-023-04559-7

**Published:** 2023-07-10

**Authors:** Jean-Louis Vincent

**Affiliations:** Department of Intensive Care, Erasme Hospital, Université Libre de Bruxelles, 1070 Brussels, Belgium

Sedative agents are among the most commonly used drugs in critical care medicine. Given the choice of agents available and the varied backgrounds of the clinicians using them, it is not surprising that individual practitioners have their own preferences. Nevertheless, it is important that the benefit/risk ratio of all therapeutic interventions should be frequently (re)assessed and use and preferences adjusted when indicated.

A meta-analysis by Kotani et al. [1], recently published in our journal, re-assessed the effects of propofol on survival. This meta-analysis included 252 randomized controlled trials (RCTs) that had compared propofol and any comparator in a total of 30,757 critically ill and perioperative patients in any clinical setting. The results suggested that propofol administration may be associated with a 10% relative increase in mortality; these findings were consistent across subgroup and sensitivity analyses.

Following publication, we received a considerable number of letters from readers who were unhappy about this publication; several, but not all, were accepted for publication. Given the strength of feeling expressed, we felt it important to briefly address the issues raised. There were several types of reaction, some more ‘visceral’ than others. Some readers clearly just disagreed with the findings and attempted to demerit the publication by any means. Others provided a more constructive critique, expressing concerns about the heterogeneity of the patient populations and the different durations of follow-up in the included trials, among others. For the ‘visceral’ reactions, we cannot do much, except acknowledge that all practitioners have preferences or even biases about their sedative of choice. It is human nature to ‘prefer’ information that supports our viewpoint and to tend to ignore or downplay other data; but the principles of scientific publishing require that all papers that are scientifically sound and relevant are made available, so that individuals have access to all data from all sides of an argument and can draw their own conclusions.

Scientific integrity and rigor are key to this process, and all questions relative to scientific quality must be taken seriously. This is the reason why we reopened the file on this article to re-read the reviewers’ comments. As for the majority of articles we receive, the reviewers had diverging opinions about the paper and stressed that its publication would raise some intense debate and discussion. We also invited an expert statistician from our editorial board to reassess the statistical analysis. After careful review, his conclusion could not be clearer: ‘I find no reason to re-evaluate the methodological and statistical review submitted initially, nor is there reason to take further action regarding the published article.’ We have therefore decided to maintain the publication as it is, with the published letters and responses from the authors [2–4], enabling our readers to make their own assessment.

Every drug has advantages and undesirable effects and, for many, this balance will vary according to multiple patient and process factors. The results of the article by Kotani et al. [1] do not mean that propofol should be abandoned. Although this meta-analysis reinforces the potential for harm already recognized more than 20 years ago [5], one must remember that every study has its limitations, and this applies to meta-analyses as well as to original contributions. Our expert statistician was reassured that ‘the discussion of Kotani et al.’s article presents healthy, and I would argue valuable, consideration of the shortcomings of meta-analysis’. This meta-analysis certainly does not constitute the final word on the safety of propofol; rather it provides one more piece of the overall research evidence. Indeed, in research there is rarely a last word, and we should always keep our minds open to new data whether or not they support our current standpoint.

## Data Availability

Not applicable.
